# Curiosity and mesolimbic functional connectivity drive information seeking in real life

**DOI:** 10.1093/scan/nsac050

**Published:** 2022-08-17

**Authors:** Kathrin C J Eschmann, Duarte F M M Pereira, Ashvanti Valji, Vera Dehmelt, Matthias J Gruber

**Affiliations:** Cardiff University Brain Research Imaging Centre (CUBRIC), School of Psychology, Cardiff University, Maindy Road, Cardiff CF24 4HQ, Wales, UK; Cardiff University Brain Research Imaging Centre (CUBRIC), School of Psychology, Cardiff University, Maindy Road, Cardiff CF24 4HQ, Wales, UK; Cardiff University Brain Research Imaging Centre (CUBRIC), School of Psychology, Cardiff University, Maindy Road, Cardiff CF24 4HQ, Wales, UK; Cardiff University Brain Research Imaging Centre (CUBRIC), School of Psychology, Cardiff University, Maindy Road, Cardiff CF24 4HQ, Wales, UK

**Keywords:** curiosity, information seeking, COVID-19, ventral tegmental area, nucleus accumbens

## Abstract

Curiosity reflects an individual’s intrinsic motivation to seek information in order to close information gaps. In laboratory-based experiments, both curiosity and information seeking have been associated with enhanced neural dynamics in the mesolimbic dopaminergic circuit. However, it is unclear whether curiosity and dopaminergic dynamics drive information seeking in real life. We investigated (i) whether curiosity predicts different characteristics of real-life information seeking and (ii) whether functional connectivity within the mesolimbic dopaminergic circuit is associated with information seeking outside the laboratory. Up to 15 months before the COVID-19 pandemic, curiosity and anxiety questionnaires and a 10-minute resting-state functional magnetic resonance imaging session were conducted. In a follow-up survey early during the COVID-19 pandemic, participants repeated the questionnaires and completed an additional questionnaire about their COVID-19-related information seeking. Individual differences in curiosity but not anxiety were positively associated with the frequency of information-seeking behaviour. Additionally, the frequency of information seeking was predicted by individual differences in resting-state functional connectivity between the ventral tegmental area and the nucleus accumbens. The present translational study paves the way for future studies on the role of curiosity in real-life information seeking by showing that both curiosity and the mesolimbic dopaminergic functional network support real-life information-seeking behaviour.

## Introduction

Curiosity—the desire to learn about specific information without any apparent extrinsic rewards—is a driver of information seeking that has been studied in a variety of laboratory settings (Gottlieb *et al.*, 2013; Kidd and Hayden, 2015; van Lieshout *et al.*, 2020). It has been proposed that curiosity can be elicited by the detection of information gaps or so-called information prediction errors (Loewenstein, 1994; Gruber and Ranganath, 2019). Subsequent information seeking serves to close these information gaps and reduce uncertainty (Gottlieb *et al.*, 2013; Gruber and Ranganath, 2019; van Lieshout *et al.*, 2021). Notably, information gaps might result in anxiety instead of curiosity if the current state of uncertainty is perceived as a threat or the necessary resources for successful uncertainty resolution are missing (Silvia, 2005; Noordewier and van Dijk, 2016; Gruber and Ranganath, 2019). Although laboratory-based research shows that curiosity is one of the key drivers of information seeking, it is unknown whether these findings translate into information seeking in everyday life, where genuine knowledge acquisition takes place. An initial hint comes from a study showing that deprivation sensitivity, that is, a subtype of curiosity reflecting the tendency to seek information in order to close information gaps, was associated with the creation of knowledge networks during the exploration of Wikipedia articles (Lydon-Staley *et al.*, 2021). Given that participants were able to explore individually chosen topics that they were interested in, it remains an open question whether curiosity drives real-life information seeking for a specific topic that is novel and personally relevant across all information seekers.

Curiosity about various types of information, such as trivia answers, magic tricks, and morbid images, has been associated with activation in the mesolimbic dopaminergic circuit (Kang *et al.*, 2009; Gruber *et al.*, 2014; Ligneul *et al.*, 2018; Duan *et al.*, 2020; Lau *et al.*, 2020; Oosterwijk *et al.*, 2020; Murphy *et al.*, 2021; Poh *et al.*, 2021). For instance, high ratings of curiosity were shown to be accompanied by increased activity in the ventral tegmental area (VTA) and the nucleus accumbens (NAcc; Gruber *et al.*, 2014), supporting the idea that the dopaminergic system supports the drive to seek information (FitzGibbon *et al.*, 2020). Consistent with this interpretation, information seeking itself has also been related to increases in activity and functional connectivity within the mesolimbic dopaminergic system (Charpentier *et al.*, 2018; Lau *et al.*, 2020). Specifically, functional connectivity between the VTA and NAcc increased during the presentation of a cue that informs about upcoming information, indicating that these dopaminergic brain regions form a network that is important for information-seeking decisions (Charpentier *et al.*, 2018). Furthermore, mesolimbic dopaminergic brain areas show task-independent intrinsic functional connectivity amongst each other and its strength varies between individuals (Kahn and Shohamy, 2013). Based on these findings, the question arises whether intrinsic functional connectivity between key regions of the mesolimbic dopaminergic circuit, such as the VTA and NAcc, is also associated with curiosity-driven information seeking in real life.

The recent coronavirus disease 2019 (COVID-19) pandemic offered a unique opportunity to study curiosity, the underlying neural dynamics in the mesolimbic dopaminergic system, and their relationship to information seeking in real life. The novelty of SARS-CoV-2 and the uncertainty about personally relevant health, social, and economic consequences introduced information gaps about COVID-19-related information to the world-wide population. Initial research has suggested that states of curiosity drive information seeking about COVID-19-related news by maximising personal utility as shown in an online experiment, in which participants’ curiosity was measured as the willingness to wait for COVID-19-related information (Abir *et al.*, 2022). However, it remains an open question whether individual differences related to how strongly people generally express curiosity, that is, curiosity traits, predict information seeking of COVID-19-related news. It is also unclear whether curiosity traits, which are thought to be rather stable personality characteristics, are unaffected by major disruptions to daily life, such as during the beginning of the COVID-19 pandemic. Based on the overarching influence of the early COVID-19 pandemic on physiological and psychological well-being (Savage *et al.*, 2020; Scrivner *et al.*, 2021), the temporal stability of curiosity traits should be especially probed under these life-changing circumstances that might be perceived as threatening and consequently enhance anxiety levels. Thus far, general anxiety levels during the COVID-19 pandemic have been associated with a reduction in the willingness to wait for information (Abir *et al.*, 2022) but also with increased information-seeking behaviour (Loosen *et al.*, 2021; Charpentier *et al.*, 2022). Anxiety predicted COVID-19-related information seeking (Charpentier *et al.*, 2022) but was not related to information seeking when obsessive compulsive behaviour was taken into account (Loosen *et al.*, 2021), indicating that anxiety is an important control variable when determining the key drivers of real-life information seeking. Altogether, it is not clear whether individual differences in curiosity solely or jointly with anxiety affect individual differences in real-life information seeking about COVID-19-related news.

**Fig. 1.**
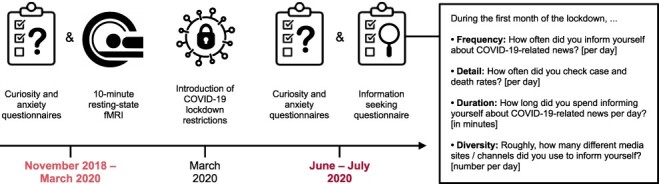
Overview of the experimental timeline and details of the COVID-19-related information seeking questionnaire. Curiosity and anxiety questionnaires as well as 10-minute resting-state functional magnetic resonance imaging (fMRI) were conducted up to 15 months before COVID-19 lockdown restrictions were introduced in the United Kingdom. During the COVID-19 pandemic, participants repeated the questionnaires and filled out an additional questionnaire about their information-seeking behaviour during the first month of lockdown. Labels for each item of the information seeking questionnaire were not presented to participants.

In the present study, participants of a previous neuroimaging study were re-invited to participate in a follow-up survey during the beginning of the COVID-19 pandemic (Figure 1). Up to 15 months before the introduction of COVID-19 lockdown restrictions, individual curiosity and anxiety scores were measured via questionnaires (Marteau and Bekker, 1992; Kashdan *et al.*, 2018) and a 10-minute resting-state functional magnetic resonance imaging (fMRI) session was conducted. After the introduction of COVID-19 lockdown restrictions, participants repeated the curiosity and anxiety questionnaires and filled out an additional questionnaire about the characteristics of their COVID-19-related information-seeking behaviour. More specifically, the information seeking questionnaire assessed the frequency, detail, duration, and diversity of information seeking during the first month of lockdown (Figure 1). We predicted that both mean and rank order of curiosity measurements should not change from before to during the COVID-19 pandemic if curiosity traits measured with the Five-Dimensional Curiosity Scale (Kashdan *et al.*, 2018) reflect temporally stable personality traits. In contrast, both mean and rank order of anxiety levels may change during the COVID-19 pandemic. Given that curiosity was shown to be a key driver of information seeking in laboratory-based experiments (Gottlieb *et al.*, 2013; Kidd and Hayden, 2015; van Lieshout *et al.*, 2020), we hypothesised that individual differences in curiosity may predict characteristics of pandemic-related information seeking in real life independently of anxiety levels. Furthermore, characteristics of information seeking should be positively associated with intrinsic functional connectivity between the VTA and NAcc, which has previously been related to curiosity and information seeking in experimental settings (Gruber *et al.*, 2014; Charpentier *et al.*, 2018).

## Materials and methods

### Participants

Overall, 62 healthy participants were invited to return to participate in this study after taking part in a previous study that included resting-state fMRI along with curiosity and anxiety measures. All participants were previously recruited from the student population of the School of Psychology at Cardiff University and received course credits and/or monetary reimbursement in return for their participation. Of those invited, 32 responded to our request, leading to a 51.61% response rate. Four participants were excluded from statistical analyses due to excess motion artefacts during fMRI data acquisition and one of them also being an outlier in the frequency, detail, and duration of information seeking as determined by the Tukey’s method with three interquartile ranges. The final sample consisted of 28 participants (2 males and 26 females, mean age  =  19.96 years, range  =  19–21 years) with normal or corrected-to-normal vision. Participants gave written informed consent and the study was approved by the Cardiff University School of Psychology Ethics Committee. Participants were reimbursed with a £10 Amazon voucher and had the chance to win an additional £50 voucher.

### Experimental design

Participants took part in two experimental sessions. In the first session, which took place before the COVID-19 pandemic, participants carried out cognitive tasks (unrelated to this study), filled out various questionnaires, and their brain activity was measured via resting-state fMRI. This session took place up to 15 months before the start of the first official UK lockdown during the COVID-19 pandemic (i.e. before 16 March 2020). In the second session, which took place during the COVID-19 pandemic, participants repeated the Five-Dimensional Curiosity Scale (Kashdan *et al.*, 2018; Cronbach’s α = 0.87) and the short version of the State-Trait Anxiety Inventory (STAI; Marteau and Bekker, 1992; Cronbach’s α = 0.79) via Qualtrics (Provo, USA). Data collection during the pandemic took place between 25 June 2020 and 7 July 2020. In addition to the questionnaires, we measured the extent to which participants carried out information-seeking behaviour specific to COVID-19-pandemic content. Specifically, the information seeking questionnaire consisted of four items, which quantified the frequency, detail, duration, and diversity of COVID-19-related information seeking per day (cf. Figure 1). For example, the item probing frequency of time spent information seeking was shown as ‘How often did you inform yourself about COVID-19-related news? [per day]’. Participants were instructed to indicate numerically for each question the amount of information seeking they carried out. Participants were instructed as such: ‘We would like to know more about how you inform yourself about the COVID-19 pandemic. Please provide a numeric answer that best fits you for the requested quantity.’

To understand the role of curiosity traits in COVID-19-related information seeking, the Five-Dimensional Curiosity Scale (Kashdan *et al.*, 2018) was administered before and during the beginning of the COVID-19 pandemic. This scale is subdivided into the subscales, joyous exploration, deprivation sensitivity, stress tolerance, social curiosity, and thrill seeking. For example, the subscale joyous exploration, which correlates with the more pleasurable, diverse motivation to seek out novel information and situations, contains items such as ‘I seek out situations where it is likely that I will have to think in depth about something.’ or ‘I enjoy learning about subjects that are unfamiliar to me.’ The subscale deprivation sensitivity which ‘is about seeking information to escape the tension of not knowing something’ (Kashdan *et al.*, 2018; p. 138) contains items such as ‘It frustrates me not having all the information I need.’ or ‘I can spend hours on a single problem because I just can’t rest without knowing the answer.’ (Kashdan *et al.*, 2018). The items were presented on a 7-point Likert scale, ranging from 1 = ‘does not describe me at all’ to 7 = ‘completely describes me’. The Five-Dimensional Curiosity Scale was selected in order to capture a broad spectrum of personality, well-being, and social factors that can influence curiosity and information seeking. In addition to this curiosity questionnaire, the short form of the STAI (Marteau and Bekker, 1992) was conducted before and during the COVID-19 pandemic. The short STAI includes items such as ‘I feel calm’, ‘I feel content’, and ‘I am worried’, and participants give the following ratings per item: 1  = ‘not at all’, 2  = ‘somewhat’, 3  = ‘moderately’, and 4  = ‘very much’.

### fMRI acquisition

Imaging data were obtained at Cardiff University Brain Research Imaging Centre, Cardiff University, using a Siemens Magnetom Prisma 3T MRI scanner with a 32-channel head coil. High-resolution T_1_-weighted structural images were obtained using an magnetisation-prepared rapid gradient-echo (MP-RAGE) sequence (repetition time (TR) = 2500 ms, echo time (TE) = 3.06 ms, flip angle = 9°, field of view (FoV) = 256 mm^2^, voxel-size = 1 mm^3^, slice thickness = 1 mm, 224 sagittal slices, bandwidth = 239 Hz/pixel, and acquisition time = 7.36 min). During the structural scan, participants watched a film in order to help reduce movement, boredom, and nervousness. For resting-state fMRI, 50 transversal slices were acquired by using an echoplanar imaging sequence (TR = 3000 ms, TE = 30 ms, flip angle = 89°, FoV 192 mm^2^, voxel-size = 2 mm^3^, slice thickness = 2 mm, bandwidth = 2170 Hz/pixel, and acquisition time = 10.11 min). A black fixation cross centred on a grey background was presented during resting-state fMRI acquisition. Participants were instructed to keep their eyes open, fixate on the cross, and try to the best of their ability to keep their minds clear. They were told not to linger on things that came to their mind (Biswal *et al.*, 1997).

### Resting-state functional connectivity pre-processing and analysis

Resting-state fMRI data were pre-processed using the functional connectivity (CONN) toolbox (version 18b; Whitfield-Gabrieli and Nieto-Castanon, 2012), in conjunction with the Statistical Parametric Mapping (SPM12) modules (Wellcome Trust Centre for Neuroimaging, London) executed in MATLAB (version 2015). In a first step, functional scans were realigned and resampled to a reference structural image using SPM12 (Andersson *et al.*, 2001) and slice-time corrected (Henson *et al.*, 1999), adjusting for differences in acquisition times between the inter-leaved scans. The Artefact Detection Tool (ART) was used to flag potential outliers with a framewise displacement > 0.5 mm and a global blood-oxygen-level-dependent (BOLD) signal change exceeding 3 SD of subject-specific means. Then, structural and functional images were normalised into Montreal Neurological Institute (MNI) space and segmented (Ashburner and Friston, 2005) with 2 mm isotropic voxels for functional images and 1 mm isotropic voxels for structural images. Functional imaging was spatially smoothed using a 6 mm full width at half maximum Gaussian Kernel. Next, images were denoised using CONN’s anatomical component noise correction procedure (Behzadi *et al.*, 2007; Chai *et al.*, 2012). Twelve noise components (three translation and three rotation parameters and their respective first-order derivatives) were identified (Friston *et al.*, 1996) to reduce motion variability in the BOLD signal. Outlier scans identified in ART were scrubbed during this step. To remove slow trends in the signal and initial magnetisation transients from the BOLD signal, a linear detrending was used. Finally, the standard band-pass filter between 0.008 and 0.09 Hz was used. In addition, data from participants with over 15% of invalid scans as identified by ART were removed from analysis. According to this exclusion criterion, data from four participants were removed.

**Fig. 2.**
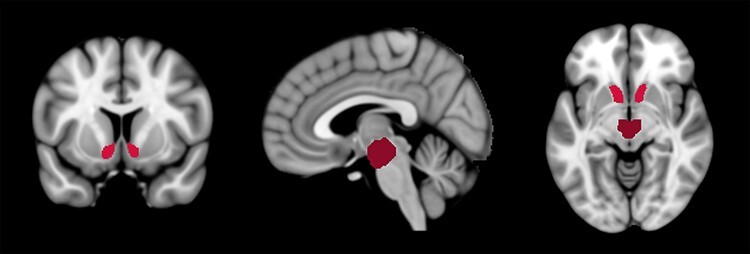
Coronal, sagittal, and axial view of the bilateral ROI masks of the VTA (dark red) and NAcc (bright red) on an MNI standard brain. The displayed ROIs were used for the resting-state functional connectivity analysis.

In order to investigate intrinsic functional connectivity between VTA and NAcc, bilateral masks of both brain regions were used as regions of interest (ROIs; Figure 2). The bilateral mask of the VTA was taken from a probabilistic atlas (Murty *et al.*, 2014) and binarised to a non-thresholded ROI mask in order to include as many VTA voxels across participants as possible. Left and right NAcc masks were taken from the Harvard–Oxford cortical atlas and combined to a bilateral NAcc mask using FMRIB Software Library (FSL) tools (Jenkinson *et al.*, 2012). The BOLD time series of each ROI was computed by averaging the voxel time series across all voxels within the ROI. Each participant’s intrinsic mesolimbic functional connectivity was computed as Fisher’s *z*-transformed bivariate Pearson correlation coefficient between the VTA and NAcc BOLD time series.

### Data analyses

Changes in curiosity and anxiety from before to during the COVID-19 pandemic were investigated using repeated-measures *t*-tests. To further understand whether the measurements are temporally stable, intraclass correlations were carried out, with the ranges <0.5, 0.5–0.75, and 0.75–0.9 indicating poor, moderate, and good temporal reliabilities, respectively (Koo and Li, 2016). To understand to what extent curiosity influences the characteristics of COVID-19-related information seeking, we calculated linear regressions. In addition, to confirm that this was not driven by underlying anxiety, we carried out a multiple regression. Finally, as we expected that these factors would contribute to real-life information seeking, we examined a potential positive relationship between curiosity and resting-state functional connectivity (RSFC) between bilateral VTA and NAcc. As such, a multiple regression was carried out to understand whether these predictors were dependent or independent of each other. For all analyses, the significance level was set to α = 0.05, and if not indicated differently, one-tailed results are reported given our directional hypotheses. In order to correct for multiple comparisons, the false discovery rate (FDR) method was applied and adjusted *P*-values were reported (Benjamini and Hochberg, 1995). In order to avoid biases from outliers for all statistical analyses, outliers were detected with the Tukey’s method using three interquartile ranges (Tukey, 1977). One participant was classified as an outlier for the frequency, detail, and duration of information seeking and was removed from analyses also due to excess motion artefacts.

## Results

### Are curiosity and anxiety stable over time?

**Fig. 3.**
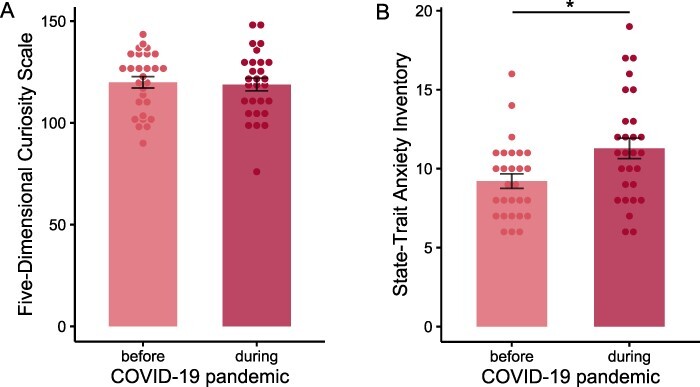
Curiosity but not anxiety scores remained stable from before to during the COVID-19 pandemic. (A) Curiosity levels measured via the Five-Dimensional Curiosity Scale remained stable over time. (B) Anxiety levels measured via the short form of the STAI increased from before to during the COVID-19 pandemic. Error bars indicate standard error of the mean.

Curiosity as measured with the Five-Dimensional Curiosity Scale (Kashdan *et al.*, 2018) did not change from before to during the COVID-19 pandemic (*t*(27) = 0.58, *P* = 0.564, *d* = 0.11, two-tailed), indicating that curiosity traits remained stable over time (Figure 3A). This was further supported by an intraclass correlation coefficient (ICC) that indicated good temporal stability of the five-dimensional curiosity scores (ICC = 0.89, 95% CI [0.80, 0.94]). Notably, participants also showed no difference from before to during the pandemic in any of the five-dimensional curiosity subscales (all FDR-adjusted *P*-values > 0.671, two-tailed) as revealed by ICC values indicating moderate to good stability for joyous exploration (ICC = 0.88, 95% CI [0.78, 0.94]), deprivation sensitivity (ICC = 0.62, 95% CI [0.29, 0.80]), stress tolerance (ICC = 0.81, 95% CI [0.65, 0.90]), social curiosity (ICC = 0.84, 95% CI [0.70, 0.92]), and thrill seeking (ICC = 0.89, 95% CI [0.79, 0.94]). In contrast, anxiety as measured by the short form of the STAI (Marteau and Bekker, 1992) increased from before to during the COVID-19 pandemic (*t*(27) = 3.57, *P* = 0.001, *d* = 0.68, two-tailed). The corresponding ICC values showed poor temporal stability (ICC = 0.48, 95% CI [0.02, 0.73]; Figure 3B). These findings suggest that even though the impact of the pandemic increased participants’ mean and rank-order anxiety levels, curiosity traits remained the same. Given that we did not find any significant differences in curiosity traits from before to during the pandemic, all subsequent analyses use curiosity trait scores measured during the pandemic in line with the measurement of information seeking of COVID-19-related news.

### Does curiosity drive real-life information seeking?

**Fig. 4.**
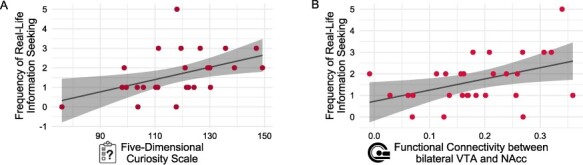
Positive relationship of curiosity and mesolimbic functional connectivity with the frequency of real-life information seeking around COVID-19-related information. (A) Five-dimensional curiosity traits measured during the pandemic were positively associated with the frequency of COVID-19-related information seeking during the first month of lockdown. (B) Resting-state functional connectivity between bilateral VTA and NAcc was positively associated with the frequency of information-seeking behaviour.

In order to investigate whether curiosity positively influences real-life information seeking, we conducted one-tailed linear regressions for each of the four characteristics captured by the information seeking questionnaire. Specifically, linear regressions with five-dimensional curiosity as predictor were calculated for the frequency, detail, duration, and diversity of COVID-19-related information seeking during the first month of lockdown as dependent variables (Figure 1). Interestingly, curiosity measured by the Five-Dimensional Curiosity Scale (Kashdan *et al.*, 2018) during the COVID-19 pandemic was positively associated with the frequency of information seeking during the first month of lockdown (β = 0.03, t(26) = 2.66, FDR-adjusted P = 0.026; Figure 4A). Thus, participants who generally experience and express high curiosity indicated by high curiosity scores informed themselves more often about COVID-19-related news per day than less curious participants. Individual differences in five-dimensional curiosity traits showed no relationship with the detail, duration, and diversity of information-seeking behaviour (all FDR-adjusted *P*-values > 0.142). Further examination of the five-dimensional curiosity subscales suggested that both joyous exploration (β = 0.10, t(26) = 2.41, FDR-adjusted P = 0.029) and deprivation sensitivity (β = 0.12, t(26) = 2.89, FDR-adjusted P = 0.019) drove the positive relationship between curiosity and frequency of information seeking when adjusted for multiple comparisons correction. The remaining subscales, stress tolerance, social curiosity, and thrill seeking, were not associated with the frequency of information seeking during the first month of lockdown (all FDR-adjusted *P*-values > 0.130). In order to assess whether the positive effects of joyous exploration and deprivation sensitivity on the frequency of information seeking were independent, both subscales were entered as predictors into a one-tailed multiple regression analysis. The multiple regression model was significant (F(2,25) = 5.25, P = 0.006) and explained 23.96% of variance. In this analysis, solely deprivation sensitivity (β = 0.09, t(25) = 2.00, P = 0.028) but not joyous exploration (β = 0.06, t(25) = 1.37, P = 0.092) predicted the frequency of real-life information seeking.

Given that anxiety during the pandemic might have positively influenced information seeking as well, we conducted one-tailed linear regressions in the same way as for the assessment of curiosity. Anxiety measured during the pandemic showed no association with the frequency, detail, duration, and diversity of information-seeking behaviour (all FDR-adjusted *P*-values > 0.141). Furthermore, we performed a multiple regression analysis to investigate whether curiosity is still positively associated with the frequency of information seeking, when anxiety is controlled for. The multiple regression model was significant (F(2,25) = 3.53, P = 0.022) and explained 15.80% of variance. According to this multiple regression, solely curiosity (β = 0.03, t(25) = 2.66, P = 0.007) but not anxiety measured during the COVID-19 pandemic (β = -0.03, t(25) = -0.44, P = 0.667) was positively associated with the frequency of real-life information-seeking behaviour (Table 1). Anxiety was also not associated with information-seeking behaviour, when the change of anxiety from before to during the COVID-19 pandemic was used as the predictor (Supplementary Material S1).

**Table 1.** Multiple regression of curiosity and anxiety measured during the COVID-19 pandemic positively predicting the frequency of real-life information seeking during the first month of lockdown

Predictor	β	SE	*t*	*P*
Constant	−1.85	1.53	−1.22	0.882
Curiosity	0.03	0.01	2.66	0.007**
Anxiety	−0.03	0.06	−0.44	0.667

Note. *P < 0.05, **P < 0.01, one-tailed.

### Does mesolimbic functional connectivity drive real-life information seeking?

In order to test whether intrinsic RSFC within the mesolimbic dopaminergic circuit is positively associated with real-life information seeking, we investigated whether RSFC between bilateral VTA and NAcc predicts different characteristics of information-seeking behaviour. One-tailed linear regressions were calculated for each measure of the information seeking questionnaire. Interestingly, functional connectivity between VTA and NAcc was positively associated with the frequency of information seeking behaviour (β = 5.20, t(26) = 2.45, FDR-adjusted P = 0.042; Figure 4B). This finding indicates that the stronger the functional connectivity within participants’ mesolimbic dopaminergic circuit, the more often participants informed themselves about COVID-19-related news during the first month of lockdown. RSFC between bilateral VTA and NAcc was not linked to the detail, duration, and diversity of COVID-19-related information-seeking behaviour (all FDR-adjusted *P*-values > 0.150), suggesting a specific relationship between mesolimbic dopaminergic RSFC and the frequency of real-life information seeking. Further exploratory analyses investigating whether mesolimbic functional connectivity is associated with individual differences in curiosity and anxiety revealed a positive relationship between VTA–NAcc RSFC and curiosity but not anxiety (Supplementary Material S2).

Given that both five-dimensional curiosity and VTA–NAcc RSFC were positively associated specifically with the frequency of COVID-19-related information seeking (Figure 4) and five-dimensional curiosity was also linked to VTA–NAcc RSFC (Supplementary Material S2), we conducted a multiple regression analysis to investigate whether curiosity trait scores and bilateral VTA–NAcc RSFC were positively associated with the frequency of information seeking dependently or independently of each other (Table 2). The multiple regression model was significant (F(2,25) = 5.39, P = 0.006) and explained 24.54% of variance. More precisely, both curiosity (β = 0.02, t(25) = 2.01, P = 0.027) and RSFC between bilateral VTA and NAcc (β = 3.75, t(25) = 1.76, P = 0.045) were independently associated with how often participants informed themselves about COVID-19-related news per day. Further analyses revealed that controlling for the delay between the resting-state fMRI scan and the behavioural measurements during the COVID-19 pandemic did not influence the aforementioned finding (Supplementary Material S3).

**Table 2.** Multiple regression of curiosity measured during the COVID-19 pandemic and RSFC between bilateral VTA and NAcc positively predicting the frequency of real-life information seeking during the first month of lockdown

Predictor	β	SE	*t*	*P*
Constant	−1.91	1.37	−1.39	0.912
Curiosity	0.02	0.01	2.01	0.027*
VTA–NAcc RSFC	3.75	2.13	1.76	0.045*

Note. *P < 0.05, **P < 0.01, one-tailed.

## Discussion

The results of the present study suggest that curiosity and mesolimbic dopaminergic functional connectivity are key drivers of information seeking in real life. Individual differences in both temporally stable curiosity traits and functional connectivity between bilateral VTA and NAcc were independently associated with the frequency of information seeking about COVD-19-related news. Thereby, the present results extend previous laboratory-based findings on information seeking (Gottlieb *et al.*, 2013; Kidd and Hayden, 2015; Charpentier *et al.*, 2018; Lau *et al.*, 2020; van Lieshout *et al.*, 2020) to real-life knowledge acquisition for a specific topic that is novel and personally relevant to all information seekers. Importantly, the role of curiosity in real-life information seeking held true when controlling for current or change in anxiety levels, which increased during the beginning of the COVID-19 pandemic but did not have an influence on real-life information seeking. This fits with the idea that specifically curiosity and the mesolimbic dopaminergic circuit play a crucial role in real-life information-seeking behaviour (Gruber and Ranganath, 2019).

The recent COVID-19 pandemic introduced high levels of uncertainty about personally relevant consequences and information gaps about COVID-19-related information. As suggested by previous research, the detection of information gaps can elicit curiosity, leading to subsequent information seeking in order to reduce uncertainty and close information gaps (Loewenstein, 1994; Gottlieb *et al.*, 2013; Gruber and Ranganath, 2019; van Lieshout *et al.*, 2021). The present results support this idea by demonstrating that individual differences in curiosity traits, that is, the general curiosity of a person, are associated with how often participants inform themselves about COVID-19-related news. Consequently, not only temporally variable curiosity states (Abir *et al.*, 2022) but also temporally stable curiosity traits are associated with COVID-19-related information seeking. The present study focused on COVID-19-related news and thus the findings cannot be generalised to other personally relevant information. We assume that an association between curiosity and information seeking would also be observed for other personally relevant news topics, but future research is needed to investigate the generalisability of the presented findings. Despite the overarching influence of the pandemic on physiological and psychological well-being (Savage *et al.*, 2020; Scrivner *et al.*, 2021), curiosity traits, as measured with the Five-Dimensional Curiosity Scale (Kashdan *et al.*, 2018), remained stable over time while anxiety levels increased from before to during the COVID-19 pandemic. This is consistent with the idea that curiosity is a rather stable personality trait that drives information-seeking behaviour in everyday life. Our finding adds to research showing that information-seeking motives remain stable over time—even during such a major impact on our lives as the COVID-19 pandemic (Kelly and Sharot, 2021). In a previous study by Lydon-Staley *et al.*, deprivation sensitivity—a subtype of curiosity that reflects the tendency to seek information in order to relieve the uncertainty created by information gaps—was associated with the creation of knowledge networks during information seeking (Lydon-Staley *et al.*, 2021). In addition, the authors found that the subscale joyous exploration—the motivation to generally seek out novel information—was linked to the variety of visited information (Lydon-Staley *et al.*, 2021). In line with these findings, the present study also showed that both deprivation sensitivity and joyous exploration predicted the frequency of real-life information seeking, but when compared to each other, it was deprivation sensitivity that drove this relationship. While these findings suggest that specific subtypes of curiosity are important drivers for real-world information seeking, future research is needed to investigate how the characteristics and content of information seeking are influenced by different curiosity subtypes.

Given that previous research found mixed results regarding the relationship between anxiety and information seeking (Loosen *et al.*, 2021; Abir *et al.*, 2022; Charpentier *et al.*, 2022), we did not have clear predictions whether curiosity alone or in addition to anxiety would be linked to real-life information seeking. While anxiety during the COVID-19 pandemic was associated with both a reduction and an increase in information-seeking behaviour in previous studies (Loosen *et al.*, 2021; Abir *et al.*, 2022; Charpentier *et al.*, 2022), anxiety was not linked to any characteristics of information seeking about COVID-19-related news in the present study. Interestingly, general anxiety and specific anxiety about the COVID-19 pandemic were suggested to have opposite effects on COVID-19-related information seeking (Abir *et al.*, 2022). Specifically, general anxiety during the COVID-19 pandemic was associated with a reduction in the willingness to wait for information whereas specific anxiety about the COVID-19 pandemic increased the willingness to wait for information (Abir *et al.*, 2022). Furthermore, no relationship between general anxiety levels and information seeking was found when controlling for obsessive compulsive behaviour (Loosen *et al.*, 2021), suggesting that it is important to take control variables into account when investigating the relationship between anxiety and information seeking. In contrast, another study that accounted for a range of control variables revealed positive relationships of both general and specific anxiety with COVID-19-related information seeking (Charpentier *et al.*, 2022). Our findings are in favour of the first of the aforementioned studies (Loosen *et al.*, 2021; Abir *et al.*, 2022), providing further evidence that general anxiety and the pandemic-induced change thereof do not increase real-life information seeking—not even in addition to curiosity scores. Thus, it seems to be specifically curiosity and mesolimbic dopaminergic functional connectivity that drive information seeking in real life. Together, these findings may inform theories that describe how anxiety and curiosity drive information seeking (Gruber and Ranganath, 2019).

In the present study, individual differences in task-independent RSFC of key brain regions of the mesolimbic dopaminergic circuit, namely bilateral VTA and NAcc, were associated with the frequency of COVID-19-related information seeking. Participants with stronger intrinsic VTA–NAcc functional connectivity informed themselves about COVID-19-related news more often than participants with weaker mesolimbic functional connectivity. Consequently, the mesolimbic dopaminergic circuit forms not only a network that supports the drive to seek information in laboratory settings (Charpentier *et al.*, 2018; FitzGibbon *et al.*, 2020) but is also important for information seeking in everyday life. In addition, individual differences in curiosity traits also showed a positive relationship with the strength of VTA–NAcc RSFC. These results extend earlier findings on dopaminergic circuit functions underlying curiosity states (Kang *et al.*, 2009; Gruber *et al.*, 2014; Ligneul *et al.*, 2018; Duan *et al.*, 2020; Lau *et al.*, 2020; Oosterwijk *et al.*, 2020; Murphy *et al.*, 2021; Poh *et al.*, 2021) by showing that curiosity traits are also associated with mesolimbic functional connectivity, suggesting that curiosity states and traits might be linked to similar neural circuits (Gruber and Ranganath, 2019; Valji *et al.*, 2019). Importantly, individual differences in curiosity traits and VTA–NAcc RSFC independently predicted the frequency of COVID-19-related information seeking. This finding suggests that curiosity and mesolimbic functional connectivity are both important drivers of real-life information seeking. Furthermore, it highlights that VTA–NAcc RSFC promotes complementary motivational processes, such as curiosity, incentive salience, or the motivation to seek new information (FitzGibbon *et al.*, 2020; Abir *et al.*, 2022). Within our recently developed Prediction, Appraisal, Curiosity, and Exploration (PACE) Framework, which lays out a series of processes to explain the neurocognitive underpinnings of curiosity, we proposed that curiosity elicits exploration and information seeking via recruitment of the mesolimbic dopaminergic circuit, in particular, the VTA and NAcc (Gruber and Ranganath, 2019). The findings of the present study support but also extend this suggestion of the PACE Framework. In line with the PACE Framework, curiosity led to increased information seeking about COVID-19-related news and was associated with enhanced VTA–NAcc functional connectivity. However, the relationship of VTA–NAcc functional connectivity with information-seeking behaviour was independent of curiosity, suggesting that mesolimbic functional connectivity in the present study might reflect complementary motivational processes that stimulate information-seeking behaviour (FitzGibbon *et al.*, 2020; Abir *et al.*, 2022). Future research is needed to better understand the precise interplay between curiosity, other motivational processes, functional connectivity within the mesolimbic dopaminergic circuit, and information seeking in order to refine the PACE Framework, accordingly. Moreover, not only the mesolimbic dopaminergic circuit but also other brain networks, such as the default mode network, should be explored because they might be important drivers of information seeking due to their role in future simulation and information integration (Ligneul *et al.*, 2018; van Lieshout *et al.*, 2018; Li *et al.*, 2019; Murphy *et al.*, 2021).

Despite the positive relationship of curiosity and mesolimbic functional connectivity with real-life information seeking, three limitations of the present study should be considered. First, even though over 50% of the invited participants took part in the survey during the COVID-19 pandemic, the sample size was relatively small and there might be a sample bias due to the observed response rate. Future studies with more participants might have more power to investigate the role of curiosity subscales in real-life information seeking. Second, the resting-state fMRI scan was conducted up to 15 months before the COVID-19 pandemic and functional connectivity might have changed over time, reflecting potentially a state-based rather than a trait-based measure. Importantly, controlling for the delay between the fMRI scan and behavioural measurements during the COVID-19 pandemic did not alter the relationship of curiosity and intrinsic mesolimbic functional connectivity with real-life information seeking and is in line with research showing short- and long-term consistency of functional connectivity measures (Damoiseaux *et al.*, 2006; Chen *et al.*, 2008; Shehzad *et al.*, 2009). Consequently, neural markers, such as RSFC, presumably reflect a trait-based rather than a state-based measure and might be used as a potential predictor of future behavioural outcomes. Third, the information seeking questionnaire in the present study assessed four characteristics of COVID-19-related information-seeking behaviour, but curiosity was specifically associated with the frequency but not with the detail, duration, and diversity of information seeking. This specificity might be explained by the fact that participants had to rate their information-seeking behaviour during the first month of lockdown in hindsight. While this might have been fairly accurate for the frequency of information seeking, participants might have misjudged the duration or diversity of their information-seeking behaviour (Parry *et al.*, 2021), making potentially the frequency the most reliable measurement. The finding that both curiosity and VTA–NAcc functional connectivity were associated specifically with the frequency of information seeking further supports the validity of the frequency measure. However, it would be fruitful if future studies might use complementary, objective measures of real-life information seeking, such as mobile tracking devices. For example, a screenomics approach, which provides a fine-grained record of an individual’s digital experience by taking screenshots (Ram *et al.*, 2020), and geolocation tracking, which has been used to show a positive relationship of dopaminergic RSFC with positive affect and variation in physical location (Heller *et al.*, 2020), seem to be promising tools.

Taken together, the present study provides evidence that curiosity and mesolimbic dopaminergic functional connectivity are key drivers of real-life information seeking, suggesting that laboratory-based findings are translatable to everyday life. Both curiosity and intrinsic functional connectivity of the mesolimbic dopaminergic circuit were positively associated with the frequency of COVID-19-related information seeking whereas anxiety levels were not linked to information seeking. Therefore, the present study paves the way for future translational studies that could address important questions about the relationship between individual differences in curiosity, intrinsic functional brain connections, and real-life information seeking. Furthermore, the present findings lay the foundation for a better understanding of individual differences in cognitive and neural characteristics that shape how individuals seek out information. A better understanding of the drivers of real-life information seeking offers the possibility to potentially help individuals who exhibit excessive or detrimental information-seeking behaviour that might negatively affect their well-being or mental health.

## Supplementary Material

nsac050_Supp

## Open practises statement

The data, on which this paper is based, and the analysis script are available at https://osf.io/dnw6p/.

## References

[R1] Abir Y., Marvin C.B., van Geen C., et al. (2022). An energizing role for motivation in information-seeking during the early phase of the COVID-19 pandemic. *Nature Communications*, 13, 2310.doi: 10.1038/s41467-022-30011-5. PMC9050882 35484153

[R2] Andersson J.L., Hutton C., Ashburner J., et al. (2001). Modeling geometric deformations in EPI time series. *NeuroImage*, 13, 903–19.doi: 10.1006/nimg.2001.0746. 11304086

[R3] Ashburner J., Friston K.J. (2005). Unified segmentation. *NeuroImage*, 26, 839–51.doi: 10.1016/j.neuroimage.2005.02.018. 15955494

[R4] Behzadi Y., Restom K., Liau J., et al. (2007). A component based noise correction method (CompCor) for BOLD and perfusion based fMRI. *NeuroImage*, 37, 90–101.doi: 10.1016/j.neuroimage.2007.04.042. 17560126 PMC2214855

[R5] Benjamini Y., Hochberg Y. (1995). Controlling the false discovery rate: A practical and powerful approach to multiple testing. *Journal of the Royal Statistical Society*, 57, 289–300.doi: 10.1111/j.2517-6161.1995.tb02031.x.

[R6] Biswal B.B., Van Kylen J., Hyde J.S. (1997). Simultaneous assessment of flow and BOLD signals in resting-state functional connectivity maps. *NMR in Biomedicine*, 10, 165–70.doi: 10.1002/(SICI)1099-1492(199706/08)10:4/5<165::AID-NBM454>3.0.CO;2-7. 9430343

[R7] Chai X.J., Castañón A.N., Ongür D., et al. (2012). Anticorrelations in resting state networks without global signal regression. *NeuroImage*, 59, 1420–8.doi: 10.1016/j.neuroimage.2011.08.048. 21889994 PMC3230748

[R8] Charpentier C.J., Bromberg-Martin E.S., Sharot T. (2018). Valuation of knowledge and ignorance in mesolimbic reward circuitry. *Proceedings of the National Academy of Sciences of the United States of America*, 115, E7255–64.doi: 10.1073/pnas.1800547115. 29954865 PMC6077743

[R9] Charpentier C.J., Cogliati Dezza I., Vellani V., et al. (2022). Anxiety increases information-seeking in response to large changes. *Scientific Reports*, 12, 7385.doi: 10.1038/s41598-022-10813-9. PMC9070976 35513397

[R10] Chen S., Ross T.J., Zhan W., et al. (2008). Group independent component analysis reveals consistent resting-state networks across multiple sessions. *Brain Research*, 1239, 141–51.doi: 10.1016/j.brainres.2008.08.028. 18789314 PMC2784277

[R11] Damoiseaux J.S., Rombouts S.A.R.B., Barkhof F., et al. (2006). Consistent resting-state networks across healthy subjects. *Proceedings of the National Academy of Sciences of the United States of America*, 103, 13848–53.doi: 10.1073/pnas.0601417103. 16945915 PMC1564249

[R12] Duan H., Fernández G., van Dongen E., et al. (2020). The effect of intrinsic and extrinsic motivation on memory formation: insight from behavioral and imaging study. *Brain Structure & Function*, 225, 1561–74.doi: 10.1007/s00429-020-02074-x. 32350643 PMC7286947

[R13] FitzGibbon L., Lau J.K.L., Murayama K. (2020). The seductive lure of curiosity: information as a motivationally salient reward. *Current Opinion in Behavioral Sciences*, 35, 21–7.doi: 10.1016/j.cobeha.2020.05.014.

[R14] Friston K.J., Williams S., Howard R., et al. (1996). Movement-related effects in fMRI time-series. *Magnetic Resonance in Medicine*, 35, 346–55.doi: 10.1002/mrm.1910350312. 8699946

[R15] Gottlieb J., Oudeyer P.-Y., Lopes M., et al. (2013). Information-seeking, curiosity, and attention: computational and neural mechanisms. *Trends in Cognitive Sciences*, 17, 585–93.doi: 10.1016/j.tics.2013.09.001. 24126129 PMC4193662

[R17] Gruber M.J., Gelman B.D., Ranganath C. (2014). States of curiosity modulate hippocampus-dependent learning via the dopaminergic circuit. *Neuron*, 84, 486–96.doi: 10.1016/j.neuron.2014.08.060. 25284006 PMC4252494

[R18] Gruber M.J., Ranganath C. (2019). How curiosity enhances hippocampus-dependent memory: The Prediction, Appraisal, Curiosity, and Exploration (PACE) Framework. *Trends in Cognitive Sciences*, 23, 1014–25.doi: 10.1016/j.tics.2019.10.003. 31706791 PMC6891259

[R19] Heller A.S., Shi T.C., Ezie C.E.C., et al. (2020). Association between real-world experiential diversity and positive affect relates to hippocampal–striatal functional connectivity, 23, 800–4.doi: 10.1038/s41593-020-0636-4. PMC9169417 32424287

[R20] Henson R.N.A., Buechel C., Josephs O., et al. (1999). The slice-timing problem in event-related fMRI. *NeuroImage*, 9, 125.

[R21] Jenkinson M., Beckmann C.F., Behrens T.E.J., et al. (2012). FSL. *NeuroImage*, 62, 782–90.doi: 10.1016/j.neuroimage.2011.09.015. 21979382

[R22] Kahn I., Shohamy D. (2013). Intrinsic connectivity between the hippocampus, nucleus accumbens, and ventral tegmental area in humans. *Hippocampus*, 23, 187–92.doi: 10.1002/hipo.22077. 23129267 PMC4118056

[R23] Kang M.J., Hsu M., Krajbich I.M., et al. (2009). The wick in the candle of learning: epistemic curiosity activates reward circuitry and enhances memory. *Psychological Science*, 20, 963–73.doi: 10.1111/j.1467-9280.2009.02402.x. 19619181

[R24] Kashdan T.B., Stiksma M.C., Disabato D.J., et al. (2018). The five-dimensional curiosity scale: Capturing the bandwitdth of curiosity and identifying four unique subgroups of curious people. *Journal of Research in Personality*, 73, 130–49.doi: 10.1016/j.jrp.2017.11.011.

[R25] Kelly C.A., Sharot T. (2021). Individual differences in information-seeking. *Nature Communications*, 12, 7062.doi: 10.1038/s41467-021-27046-5. PMC8642448 34862360

[R26] Kidd C., Hayden B.Y. (2015). The psychology and neuroscience of curiosity. *Neuron*, 88, 449–60.doi: 10.1016/j.neuron.2015.09.010. 26539887 PMC4635443

[R27] Koo T.K., Li M.Y. (2016). A guideline of selecting and reporting intraclass correlation coefficients for reliability research. *Journal of Chiropractic Medicine*, 15, 155–63.doi: 10.1016/j.jcm.2016.02.012. 27330520 PMC4913118

[R28] Lau J.K.L., Ozono H., Kuratomi K., et al. (2020). Shared striatal activity in decisions to satisfy curiosity and hunger at the risk of electric shocks. *Nature Human Behaviour*, 4, 531–43.doi: 10.1038/s41562-020-0848-3. 32231281

[R29] Li Y., Huo T., Zhuang K., et al. (2019). Functional connectivity mediates the relationship between self-efficacy and curiosity. *Neuroscience Letters*, 711, 134442.doi: 10.1016/j.neulet.2019.134442. 31442514

[R30] Ligneul R., Mermillod M., Morisseau T. (2018). From relief to surprise: dual control of epistemic curiosity in the human brain. *NeuroImage*, 181, 490–500.doi: 10.1016/j.neuroimage.2018.07.038. 30025853

[R31] Loewenstein G. (1994). The psychology of curiosity: A review and reinterpretation. *Psychological Bulletin*, 116, 75–98.doi: 10.1037/0033-2909.116.1.75.

[R32] Loosen A.M., Skvortsova V., Hauser T.U. (2021). Obsessive-compulsive symptoms and information seeking during the Covid-19 pandemic. *Translational Psychiatry*, 11, 309.doi: 10.1038/s41398-021-01410-x. PMC8138954 34021112

[R33] Lydon-Staley D.M., Zhou D., Blevins A.S., et al. (2021). Hunters, busybodies and the knowledge network building associated with deprivation curiosity. *Nature Human Behaviour*, 5, 327–36.doi: 10.1038/s41562-020-00985-7. PMC8082236 33257879

[R34] Marteau T.M., Bekker H. (1992). The development of a six‐item short‐form of the state scale of the Spielberger State—Trait Anxiety Inventory (STAI). *The British Journal of Clinical Psychology*, 31, 301–6.doi: 10.1111/j.2044-8260.1992.tb00997.x. 1393159

[R35] Murphy C., Ranganath C., Gruber M.J. (2021). Connectivity between the hippocampus and default mode network during the relief – but not elicitation – of curiosity supports curiosity-enhanced memory enhancements. *bioRxiv*.doi: 10.1101/2021.07.26.453739.

[R36] Murty V.P., Shermohammed M., Smith D.V., et al. (2014). Resting state networks distinguish human ventral tegmental area from substantia nigra. *NeuroImage*, 100, 580–9.doi: 10.1016/j.neuroimage.2014.06.047. 24979343 PMC4370842

[R37] Noordewier M.K., van Dijk E. (2016). Interest in complex novelty. *Basic and Applied Social Psychology*, 38, 98–110.doi: 10.1080/01973533.2016.1153474.

[R38] Oosterwijk S., Snoek L., Tekoppele J., et al. (2020). Choosing to view morbid information involves reward circuitry. *Scientific Reports*, 10, 15291.doi: 10.1038/s41598-020-71662-y. PMC7499173 32943668

[R39] Parry D.A., Davidson B.I., Sewall C.J.R., et al. (2021). A systematic review and meta-analysis of discrepancies between logged and self-reported digital media use. *Nature Human Behaviour*, 5, 1535–47.doi: 10.1038/s41562-021-01117-5. 34002052

[R40] Poh J.-H., Vu M.-A.T., Stanek J.K., et al. (2021). Tuned to learn: An anticipatory hippocampal convergence state conducive to memory formation revealed during midbrain activation. *bioRxiv*.doi: 10.1101/2021.07.15.452391.

[R41] Ram N., Yang X., Cho M.-J., et al. (2020). Screenomics: A new approach for observing and studying individuals' digital lives. *Journal of Adolescent Research*, 35, 16–50.doi: 10.1177/0743558419883362. 32161431 PMC7065687

[R42] Savage M.J., James R., Magistro D., et al. (2020). Mental health and movement behaviour during the COVID-19 pandemic in UK university students: Prospective cohort study. *Mental Health and Physical Activity*, 19, 100357.doi: 10.1016/j.mhpa.2020.100357.

[R43] Scrivner C., Johnson J.A., Kjeldgaard-Christiansen J., et al. (2021). Pandemic practice: Horror fans and morbidly curious individuals are more psychologically resilient during the COVID-19 pandemic. *Personality and Individual Differences*, 168, 110397.doi: 10.1016/j.paid.2020.110397. PMC7492010 32952249

[R44] Shehzad Z., Kelly A.M.C., Reiss P.T., et al. (2009). The resting brain: Unconstrained yet reliable. *Cerebral Cortex*, 19, 2209–29.doi: 10.1093/cercor/bhn256. 19221144 PMC3896030

[R45] Silvia P.J. (2005). What is interesting? Exploring the appraisal structure of interest. *Emotion*, 5, 89–102.doi: 10.1037/1528-3542.5.1.89. 15755222

[R46] Tukey J.W. (1977). *Exploratory Data Analysis*. Addison-Wesley. Reading, MA, USA.

[R47] Valji A., Priemysheva A., Hodgetts C.J., et al. (2019). White matter pathways supporting individual differences in epistemic and perceptual curiosity. *bioRxiv*.doi: 10.1101/642165.

[R48] van Lieshout L.L.F., Vandenbroucke A.R.E., Müller N.C.J., et al. (2018). Induction and relief of curiosity elicit parietal and frontal activity. *The Journal of Neuroscience*, 38, 2579–88.doi: 10.1523/JNEUROSCI.2816-17.2018. 29439166 PMC6705901

[R49] van Lieshout L.L.F., de Lange F.P., Cools R. (2020). Why so curious? Quantifying mechanisms of information seeking. *Current Opinion in Behavioral Sciences*, 35, 112–7.doi: 10.1016/j.cobeha.2020.08.005.

[R50] van Lieshout L.L.F., Traast I.J., de Lange F.P., et al. (2021). Curiosity or savouring? Information seeking is modulated by both uncertainty and valence. *PLOS ONE*, 16, e0257011.doi: 10.1371/journal.pone.0257011. PMC8462690 34559816

[R51] Whitfield-Gabrieli S., Nieto-Castanon A. (2012). *Conn*: A functional connectivity toolbox for correlated and anticorrelated brain networks. *Brain Connectivity*, 2, 125–41.doi: 10.1089/brain.2012.0073. 22642651

