# Supplementary material for: Curiosity and mesolimbic functional connectivity drive information seeking in real life
Source: Soc Cogn Affect Neurosci. 2022 Aug 17;18(1):nsac050. doi: 10.1093/scan/nsac050 (PMC9452113; doi:10.1093/scan/nsac050)
Supplement: nsac050_Supp [file nsac050_supp.zip › Supplementary Material.pdf]

Supplementary Material

S1. Is the change in anxiety from before to during the COVID-19 pandemic associated with real-life information seeking?

It is conceivable that the change in anxiety from before to during the COVID-19 pandemic reflects a more fine-grained measurement of anxiety induced by the COVID-19 pandemic than anxiety measured during the COVID-19 pandemic. The change in anxiety from before to during the COVID-19 pandemic was calculated as the difference between anxiety scores (anxiety during – anxiety before). All regression analyses including anxiety as a predictor of real-life information seeking were therefore conducted with the change in anxiety instead of anxiety measured during the COVID-19 pandemic. One-tailed linear regressions revealed that the change in anxiety from before to during the COVID-19 pandemic was not associated with the frequency, detail, duration, and diversity of information-seeking behaviour (all FDR-adjusted p-values > .399). Furthermore, the multiple regression model including both curiosity and change in anxiety as predictors of information seeking was significant ($F(2,25) = 4.24$, $p = .013$) and explained 19.36% of variance (Table S1). Importantly, curiosity still positively predicted the frequency of information seeking (β = 0.03, $t(25) = 2.75$, $p = .005$) while the effect of the change in anxiety on the frequency of information seeking remained non-significant (β = -0.07, $t(25) = -1.14$, $p = .868$).

Table S1

Multiple regression testing whether curiosity and the change in anxiety from before to during the COVID-19 pandemic positively predicts the frequency of real-life information seeking during the first month of lockdown.

Predictor	β	*SE*	*t*	*p*
Constant	-2.03	1.41	-1.44	.918
Curiosity	0.03	0.01	2.75	.005**
Change in Anxiety	-0.07	0.06	-1.14	.868

Note. * $p < .05$, ** $p < .01$, one-tailed.

S2. Is mesolimbic functional connectivity associated with curiosity and anxiety?

In order to explore whether resting-state functional connectivity between bilateral VTA and NAcc predicted later curiosity during the COVID-19 pandemic, we conducted a one-tailed linear regression. Bilateral VTA-NAcc RSFC predicted curiosity measured during the COVID-19 pandemic (β = 59.31, $t(26) = 1.82$, $p = .040$), indicating that mesolimbic functional connectivity is linked to curiosity (Figure S1). This relationship was not driven by any of the five-dimensional curiosity subscales when adjusted for multiple comparisons (all FDR-adjusted p-values > .165).

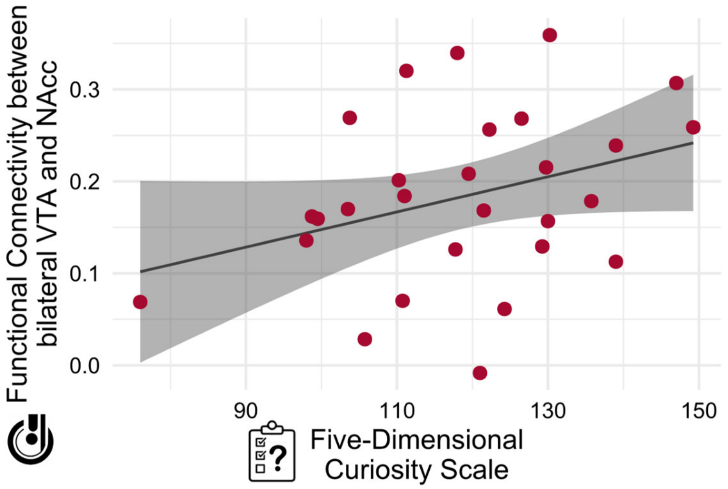

Figure S1. Positive relationship mesolimbic functional connectivity with later curiosity. Resting-state functional connectivity between bilateral VTA and NAcc was positively associated with curiosity measured during the COVID-19 pandemic.

The same one-tailed linear regression was conducted separately for anxiety measured during the COVID-19 pandemic and the change in anxiety from before to during the pandemic. Functional connectivity between bilateral VTA and NAcc predicted neither anxiety during the COVID-19 pandemic (β = -1.66, $t(26) = -0.23$, $p = .591$) nor the change in anxiety from before to during the COVID-19 pandemic (β = 0.17, $t(26) = 0.03$, $p = .489$). Hence, mesolimbic functional connectivity was not linked to anxiety or the pandemic-induced change thereof.

S3. Does the delay between the resting-state fMRI scan and the survey during the COVID-19 pandemic influence the observed relationships?

The average delay between the resting-state fMRI scan and the survey during the COVID-19 pandemic was *M* = 479.57 days (*SD* = 156.05 days), ranging from 106 to 601 days. We included the delay as a predictor of no interest in the multiple regression analysis that included curiosity and VTA-NAcc functional connectivity (cf. Table 2). The multiple regression model was significant ($F(3,24) = 3.48$, $p = .016$) and explained 21.57% of variance (cf. Table 2). Importantly, both curiosity ($\beta = 0.02$, $t(24) = 1.91$, $p = .034$) and VTA-NAcc functional connectivity ($\beta = 3.72$, $t(24) = 1.71$, $p = .0499$) still remained independently associated with how often participants informed themselves about COVID-19-related news per day (Table S2). Consequently, including the delay between the resting-state fMRI scan and behavioural measures during the COVID-19 pandemic as a control variable into the regression analyses did not change the observed findings.

Table S2

Multiple regression of curiosity measured during the COVID-19 pandemic and resting-state functional connectivity (RSFC) between bilateral VTA and NAcc positively predicting the frequency of real-life information seeking during the first month of lockdown, while controlling for the delay between fMRI scan and behavioural measurements.

Predictor	β	*SE*	*t*	*p*
Constant	-1.71	1.65	-1.04	.857
Delay	-0.01	0.01	-0.23	.555
Curiosity	0.02	0.01	1.91	.034*
VTA-NAcc RSFC	3.72	2.17	1.71	.0499*

Note. * $p < .05$, ** $p < .01$, one-tailed.
